# Silent Diabetes: Key Risk Factors Among the Low-Income Population of Langkawi Island, Kedah, Malaysia (2022-2023)

**DOI:** 10.7759/cureus.71386

**Published:** 2024-10-13

**Authors:** Syuaib Aiman Amir Kamarudin, Afiq Izzudin A Rahim, Muhd Suhaili Muhd Shueib, Mansor Ismail

**Affiliations:** 1 Department of Community Medicine, Universiti Sains Malaysia School of Medical Sciences, Kota Bharu, MYS; 2 Langkawi District Health Office, Kedah Health State Department, Langkawi, MYS; 3 Langkawi Health District Office, Kedah Health State Department, Langkawi, MYS

**Keywords:** diabetes, geographical isolation, langkawi island, low income population, non-communicable disease (ncds), northern malaysia, obesity, public health policy, undiagnosed diabetes

## Abstract

Background: Diabetes mellitus (DM) poses a growing global health challenge, contributing to significant morbidity, mortality, and economic burden. In Malaysia, the prevalence of diabetes is increasing, especially among low-income populations with limited access to healthcare. Many cases remain undiagnosed, increasing the risk of severe complications and further straining healthcare resources. Island populations, such as those in Langkawi, are particularly vulnerable due to geographical isolation, socioeconomic constraints, and inadequate healthcare services.

Objectives: To determine the prevalence of undiagnosed DM and identify the associated risk factors among the low-income population on Langkawi Island, Kedah, Malaysia.

Methods: We conducted a cross-sectional study from January 2022 to December 2023, involving 1,070 participants aged 40 years and above, all eligible under the low-income scheme. Data on sociodemographic characteristics, body mass index (BMI), lifestyle, and medical history were collected through a structured proforma from four local health clinics. Logistic regression analysis was used to identify significant predictors of undiagnosed DM. The model's predictive accuracy was assessed using the area under the receiver operating characteristic (ROC) curve.

Results: The prevalence of undiagnosed DM among the low-income population on Langkawi Island was 6.7%. Multiple logistic regression found three important predictors: having a higher BMI (overweight: adjusted odds ratio (AOR): 2.72; 95% confidence interval (CI): 1.40-5.30; p = 0.003; obese: AOR: 2.43; 95% CI: 1.19-5.00; p = 0.015); living on a smaller island (AOR: 1.71; 95% CI: 1.03-2.85; p = 0.039); and having a medical history (AOR: 0.21; 95% CI: 0.12-0.36; p < 0.001). The model demonstrated good predictive accuracy with an area under the ROC curve of 0.758 and correctly classified 93.3% of cases.

Conclusion: This study reveals a significant burden of undiagnosed DM within Langkawi's low-income population, especially among individuals with higher BMI and those residing in geographically isolated areas. The findings highlight the urgent need for enhanced, context-specific screening programs and early detection efforts tailored to this vulnerable population. Effective public health strategies should prioritize regular health check-ups, obesity management, and improved access to healthcare services in isolated communities to reduce the prevalence and complications associated with undiagnosed diabetes.

## Introduction

Diabetes mellitus (DM) is an escalating public health crisis with significant global implications. The disease affects millions worldwide, placing a substantial burden on healthcare systems, economies, and individuals [1]. The International Diabetes Federation (IDF) predicts that 783 million adults worldwide will have DM by 2045, with a disproportionate impact on low- and middle-income countries (LMICs) [2]. These countries are home to most people with DM, largely due to rapid urbanization, lifestyle changes, and limited access to healthcare services. Malaysia, mirroring global trends, is witnessing a troubling rise in the prevalence of DM, particularly among its socio-economically disadvantaged populations. The National Health and Morbidity Survey (NHMS) 2019 revealed that nearly 20% of Malaysian adults suffer from DM, underscoring the urgent need for effective public health interventions to manage and mitigate the disease's impact [3].

The high prevalence of undiagnosed cases further complicates the challenge of managing DM. Globally, an estimated 44.7% of adults with DM are unaware of their condition, leading to delayed treatment and an increased risk of severe complications, including cardiovascular disease, neuropathy, and retinopathy [4]. LMICs bear a particularly severe burden of undiagnosed DM, accounting for over 87.5% of cases [2]. Island populations, confronting unique challenges like geographical isolation, limited healthcare access, and significant socioeconomic barriers, exacerbate this issue. These factors contribute to the difficulty of early detection and management of DM, making populations like those on Langkawi Island particularly vulnerable. Langkawi, a popular tourist destination in Malaysia, is home to a large proportion of the low-income, or B40 population, the bottom 40% of income earners in the country. The island's distinct geographical and socioeconomic context presents additional hurdles to healthcare access, rendering the population susceptible to undiagnosed chronic conditions like DM. Previous studies have consistently shown that residents of island communities are more likely to have undiagnosed DM due to factors such as reduced healthcare access, lower health literacy, and a reliance on traditional or informal healthcare practices [5-7]. Understanding the epidemiology of undiagnosed DM in such settings is crucial for developing targeted interventions aimed at improving healthcare access and outcomes for these vulnerable populations.

Literature has shown that multiple factors contribute to the prevalence of undiagnosed DM in low-income populations, such as age, body mass index (BMI), and geographical location [8,9]. In Malaysia, undiagnosed DM has been linked to sociodemographic factors like age, gender, and education level, alongside health-related factors, including BMI, physical inactivity, and family history of DM [8,10]. This pattern is consistent globally. For instance, research in Ethiopia found that low physical activity and family history were strongly associated with undiagnosed DM [11]. Obesity has also been consistently highlighted as a major risk factor, with obese individuals more likely to remain undiagnosed [12]. Geographical isolation, particularly in island populations with limited healthcare access, further exacerbates these risks [13]. For example, adults from the Marshall Islands living on remote islands exhibited a higher prevalence of undiagnosed DM [5]. Moreover, undiagnosed DM significantly increases the risk of complications such as cardiovascular disease, neuropathy, and nephropathy, reducing quality of life and elevating mortality rates [14,15].

Given the significant public health implications of undiagnosed DM, particularly in vulnerable populations, this study aims to address the existing gaps in the literature by examining the factors associated with undiagnosed DM among the low-income population on Langkawi Island. This population's unique healthcare challenges, including geographical isolation and limited healthcare infrastructure, necessitate a focused approach to understanding the prevalence and determinants of undiagnosed DM. By identifying the key factors contributing to the high prevalence of undiagnosed DM in this setting, the study seeks to contribute to the broader goal of reducing health disparities and improving the quality of life for low-income populations in Malaysia.

## Materials and methods

We conducted this cross-sectional study to evaluate the factors associated with undiagnosed DM among the low-income population residing on Langkawi Island, Malaysia. The study focused on individuals eligible for the national low-income scheme, which targets the bottom 40% of income earners in Malaysia. The inclusion criteria required participants to be aged 40 years and older, as this demographic is at a higher risk for developing DM and related complications. The study period was conducted from January 2022 to December 2023, during which the eligible population underwent routine health screenings under the low-income scheme program. 

Ethical approval was obtained from the National Medical Research Register (NMRR) of the Ministry of Health, Malaysia (Ref: 23-03345-NGN) and the Human Research Ethics Committee (HREC) of Universiti Sains Malaysia (Study Protocol Code: USM/JEPeM/KK/23110859) before the study commenced.

The sample size was calculated using the single proportion formula with a 95% confidence interval, initially requiring 974 participants to ensure adequate statistical power. To account for potential issues such as missing data or outliers, we adjusted the sample size by 10%, resulting in an estimated 1,082 participants. However, 1,070 individuals completed the screening and met the inclusion criteria. Hence, no sampling technique was applied, as all eligible participants were included to maximize data validity.

Data were collected using a structured proforma checklist specifically designed to extract key sociodemographic, lifestyle, and health-related information from the low-income registry at four Langkawi Health Clinics. Essential variables included age, gender, BMI, employment status, physical activity, smoking and alcohol consumption, medical history, family history of DM, and geographical location. The metabolic equivalent of task (MET) was used to measure physical activity. Participants were either classified as active (≥1500 MET/3 days of vigorous activity or ≥3000 MET/week with any activity) or not active. The checklist ensured a standardized and consistent approach to data collection, maintaining high data quality and reliability across participants.

We analyzed the data using IBM SPSS Statistics for Windows, version 28 (IBM Corp., Armonk, NY), a widely used statistical software in medical research. Descriptive statistics summarised the sociodemographic characteristics, lifestyle factors, and health status of the study population, providing an overview and highlighting patterns in the data. To calculate the prevalence of undiagnosed DM, we expressed the number of undiagnosed cases as a percentage of the total study population. This metric provided a clear indication of the extent of undiagnosed DM within the low-income population of Langkawi Island.

Next, simple logistic regression was used for univariable analysis to assess the association between each independent variable and undiagnosed DM. Variables with a p-value < 0.25 were included in the multivariable analysis to avoid excluding potentially important factors. This approach allowed for a more comprehensive model by considering possible interactions and confounding effects. To control for confounding factors and determine the independent predictors of undiagnosed DM, we performed multiple logistic regression. This advanced statistical technique allowed us to adjust for the effects of multiple variables simultaneously, providing a more accurate estimation of the associations. We presented the results as adjusted odds ratios (AOR) with 95% confidence intervals (CI), thereby quantifying the strength and direction of the associations. We evaluated the model fitness of the final logistic regression model using the Hosmer-Lemeshow test, which assesses whether the observed outcomes match the expected outcomes predicted by the model. A non-significant p-value from this test indicated that the model was a satisfactory fit for the data. Additionally, we assessed the model’s discriminatory power using the receiver operating characteristic (ROC) curve, which plots the true positive rate against the false positive rate. The area under the ROC curve (AUC) was calculated to determine the accuracy of the model in distinguishing between participants with and without undiagnosed DM.

## Results

Socio-demographic and health characteristics

The study population consisted of 1,070 participants, with a mean age of 58.7 years (SD = 10.9), and predominantly female (60.1%). The majority were married (78.5%) and had completed primary (48.6%) or secondary (40.7%) education, with only 8.5% having no formal education and 2.1% with tertiary education. Regarding employment, 44.9% were employed, and 55.1% were unemployed.

In terms of health status, 36.2% of participants were classified as overweight and 26.8% as obese, with only 33.2% in the normal BMI range. The prevalence of abnormal mental health status was 25.9%. A significant portion of the participants (61.1%) reported a medical history, and 31.3% had a family history of diabetes. Geographically, 27.0% of participants resided on smaller islands, compared to 73.0% on the main island.

These characteristics underscore the study's focus on a predominantly older, overweight, and socioeconomically disadvantaged population. The high prevalence of overweight and obesity, along with geographic isolation and limited healthcare access, reinforces the need for targeted diabetes screening and intervention programs in this population.

Factors associated with undiagnosed DM

The analysis of factors associated with undiagnosed DM used simple and multiple logistic regression models. Simple logistic regression, as highlighted in Table 1, identified several significant variables. People with a medical history were more likely to have undiagnosed DM (Crude OR = 5.27, 95% CI: 3.04-9.13, p < 0.001). People who lived on smaller islands (Crude OR = 2.30, 95% CI: 1.40-3.77, p = 0.001) and people who were unemployed (Crude OR = 2.30, 95% CI: 1.40-3.77, p = 0.001) were also more likely to have it.

**Table 1:** Simple logistic regression analysis of associated factors undiagnosed DM among low-income population populations on Langkawi Island (n=1070) * median; B^1^: Crude Coefficient Regression; DM: diabetes mellitus; OR: odds ratio

Variables	Undiagnosed DM		B^1^	Crude OR (95% CI)	p-value
	Yes, n = 72 (6.7%)	No, n= 998 (93.3%)			
Age (years)*	54.0	58.0	-0.033	0.968 (0.945,0.991)	0.007
Gender					
Male	29 (40.3)	398 (39.9)	-0.017	0.947 (0.604,1.602)	0.947
Female	43 (59.7)	600 (60.1)		Ref	
Marital status					
Married	63 (87.5)	777 (77.9)	-0.689	0.502 (0.256,1.026)	0.059
Unmarried	9 (12.5)	221 (22.1)		Ref	
Employment status					
Yes	46 (63.9)	434 (43.5)		Ref	
No	26 (36.1)	564 (56.5)	0.833	2.299 (1.399,3.779)	0.001
Geographical area					
Main island	39 (54.2)	742 (74.3)	-0.897	Ref	
Small island	33 (45.8)	256 (25.7)		0.408 (0.251,0.662)	<0.001
Education level					
No education	5 (6.9)	86 (8.6)	-0.333	0.717 (0.093,5.528)	0.749
Primary	40 (55.6)	480 (48.1)	-0.087	0.917 (0.342,2.455)	0.863
Secondary	26 (36.1)	410 (41.1)	0.273	1.314 (0.788,2.191)	0.295
Tertiary	1 (1.4)	22 (2.2)		Ref	
Medical history					
Yes	18 (25)	636 (63.7)	1.662	5.271 (3.045,9.125)	<0.001
No	54 (75)	362 (36.3)		Ref	
BMI					
Underweight	2 (2.8)	39 (3.9)	0.299	1.349 (0.294,6.200)	0.700
Normal	13 (18.1)	342 (34.3)		Ref	
Overweight	35 (48.6)	352 (35.3)	0.962	2.616 (1.360,5.030)	0.004
Obese	22 (30.6)	265 (26.6)	0.781	2.184 (1.080,4.416)	0.030
Mental status					
Normal	50 (69.4)	743 (74.4)		Ref	
Abnormal	22 (30.6)	255 (25.6)	-0.248	0.780 (0.463,1.314)	0.350
Physical activities					
Active	54 (75)	800 (80.2)		Ref	
Inactive	18(25)	198 (19.8)	-0.298	0.743 (0.426,1.294)	0.294
Smoking status					
Yes	7 (9.7)	138 (13.8)	0.399	1.490 (0.669,3.317)	0.329
No	65 (90.3)	860 (86.2)		Ref	
Alcohol status					
Yes	1 (1.4)	12 (1.2)	-0.146	0.864 (0.111,6.740)	0.889
No	71 (98.6)	986 (98.8)		Ref	
Family history					
Yes	18 (25)	317 (31.8)	0.334	1.396 (0.806,2.420)	0.234
No	54 (75)	681 (68.2)		Ref	

Age was negatively associated with undiagnosed DM (Crude OR = 0.97, 95% CI: 0.95-0.99, p = 0.007), indicating younger participants were more likely to have undiagnosed DM. Overweight (Crude OR = 2.62, 95% CI: 1.36-5.03, p = 0.004) and obese (Crude OR = 2.18, 95% CI: 1.08-4.42, p = 0.030) participants also had a higher likelihood compared to those with normal BMI.

Other factors such as gender, marital status, education, mental status, physical activity, smoking, alcohol use, and family history were not significantly associated with undiagnosed DM.

Table 2 shows that, after adjusting for confounders, three variables remained significant predictors of undiagnosed DM. BMI was a key factor, with overweight individuals 2.72 times more likely to have undiagnosed DM (AOR = 2.72, 95% CI: 1.40-5.30, p = 0.003) and obese individuals 2.43 times more likely (AOR = 2.43, 95% CI: 1.19-5.00, p = 0.015). Geographical location also remained significant, with residents of smaller islands 71% more likely to have undiagnosed DM compared to those on the main island (AOR = 1.71, 95% CI: 1.03-2.85, p = 0.039). Lastly, medical history was inversely associated with undiagnosed DM, as individuals with a documented medical history were significantly less likely to have undiagnosed DM (AOR = 0.21, 95% CI: 0.12-0.36, p < 0.001). These findings underscore the critical role of BMI, geographical isolation, and healthcare access in identifying and managing undiagnosed DM in low-income populations on Langkawi Island.

**Table 2:** Multiple logistic regression (LR) analysis of associated factors undiagnosed DM among low-income population populations on Langkawi Island (n=1070) Constant = -2.802; backward LR method applied; no multicollinearity and no interaction; Hosmer and Lemeshow test; p-value = 0.508; classification table 93.3% correctly classified; area under receiver operating characteristics (ROC) = 75.8% B^1^: Adjusted Coefficient Regression; DM: diabetes mellitus; OR: odds ratio

Variables	Undiagnosed DM		B^1^	Adjusted OR (95% CI)	p-value
	Yes, n = 72 (6.7%)	No, n = 998 (93.3%)			
BMI					
Underweight	2 (2.8)	39 (3.9)	-0.154	0.857 (0.183,4.020)	0.845
Normal	13 (18.0)	342 (34.3)		Ref	
Overweight	35 (48.6)	352 (35.3)	1.001	2.720 (1.396,5.302)	0.003
Obese	22 (30.6)	265 (26.5)	0.889	2.434 (1.185,4.999)	0.015
Geographical area					
Main island	39 (54.2)	742 (74.3)		Ref	
Small island	33 (45.8)	256 (25.7)	0.537	1.710 (1.027,2.848)	0.039
Medical history					
No	18 (25)	636 (63.7)		Ref	
Yes	54 (75)	362 (36.3)	-1.584	0.205 (0.116,0.362)	<0.001

The model's sensitivity was evaluated using the ROC curve, which yielded an AUC of 0.758, indicating good discriminatory ability. The Hosmer-Lemeshow test showed a p-value of 0.508, confirming the model's fit to the data. Additionally, the classification table demonstrated that 93.3% of cases were correctly classified, reinforcing the model's accuracy. No multicollinearity was observed among the predictors, and no significant interactions were identified, confirming the independence of the variables. These results highlight the model's reliability in identifying key factors associated with undiagnosed DM in this population.

## Discussion

The prevalence of undiagnosed DM in the low-income population on Langkawi Island, identified at 6.7%, remains a significant public health concern despite being slightly lower than the 8.9% reported in the NHMS 2019 for the broader Malaysian population [3]. However, during the recent NHMS 2023, the prevalence of undiagnosed DM is lowering at 5.9% [16]. The specific characteristics of the study population, including the island’s geographical isolation and potentially improved access to local health interventions, may account for this lower prevalence. However, the figure is still alarming, particularly given the healthcare access challenges that are prevalent in isolated island communities. These challenges frequently result in delayed diagnosis and management of chronic diseases, including diabetes. As with other studies done on island populations, our results are in line with reported rates of undiagnosed diabetes. This shows that these groups face special risks [5].

Undiagnosed DM is particularly concerning due to its association with severe complications, including cardiovascular diseases, neuropathy, retinopathy, and nephropathy, which contribute to increased mortality rates and deteriorated quality of life [14,15]. The early identification of undiagnosed cases is therefore crucial for implementing timely and effective interventions to mitigate these risks, prevent complications, and reduce the overall healthcare burden.

The multivariable analysis in this study identified three significant predictors of undiagnosed DM: BMI, geographical area, and no medical history. These findings are crucial as they provide an accurate assessment of the factors that independently contribute to the risk of undiagnosed diabetes by controlling for potential confounding variables. BMI emerged as a particularly important factor, with overweight and obese individuals significantly more likely to have undiagnosed DM. The study found that people who are overweight or obese had an AOR of 2.72 and people who are obese had an AOR of 2.43. This means that people in these weight categories are about 2.7 and 2.4 times more likely to have DM without knowing it than people whose BMI is normal. This finding is supported by a substantial body of literature linking obesity with an increased risk of type 2 DM [8,17,18]. Obesity contributes to insulin resistance, a primary mechanism in the pathogenesis of type 2 DM [10]. The study’s findings suggest that individuals with a higher BMI are at greater risk of remaining undiagnosed, possibly due to the subtlety of symptoms or infrequent medical consultations. Therefore, public health strategies aimed at weight management and obesity prevention are critical to reducing the incidence of undiagnosed DM, particularly in populations where obesity is prevalent.

Geographical isolation was another significant factor identified in the analysis, with individuals residing on smaller islands having a higher likelihood of undiagnosed DM compared to those living on the main island. In this study, we had an AOR of 1.71, indicating a 71% increase in the risk of undiagnosed DM for those on smaller islands. This finding highlights the unique challenges faced by geographically isolated populations, where limited access to healthcare services can result in delayed diagnosis and treatment of chronic conditions such as diabetes. The broader literature supports this finding, showing that residents of rural and isolated areas are at greater risk of undiagnosed and untreated health conditions due to barriers such as fewer healthcare facilities, lower availability of healthcare professionals, and reduced health-seeking behavior [6,19,20]. Addressing these disparities requires targeted interventions to improve healthcare access in isolated communities. Mobile health units that bring healthcare services directly to these communities have shown effectiveness in improving access to care. Telemedicine services, which allow patients to consult with healthcare providers remotely, are also essential for overcoming geographical barriers to healthcare access. A study done among US populations shows that patients significantly reduced use of preventive and elective use and increased use of telemedicine during the COVID-19 pandemic [21]. Additionally, community-based health education programs that promote regular health screenings and raise awareness about the importance of early detection of chronic conditions like DM are critical.

Interestingly, the study also found an inverse relationship between having a documented medical history and the likelihood of undiagnosed DM. Participants with a documented medical history were significantly less likely to have undiagnosed DM, with an AOR of 0.21, suggesting that these individuals were about 80% less likely to have undiagnosed diabetes compared to those without a medical history. This finding underscores the importance of regular medical check-ups and healthcare interactions in the early detection and management of DM. Regular interactions with the healthcare system increase the likelihood of early detection of chronic conditions like DM, as healthcare providers are more likely to screen for such conditions even in the absence of symptoms [22]. The relationship between a documented medical history and the likelihood of undiagnosed DM emphasizes the role of continuous medical care and regular health screenings in reducing the prevalence of undiagnosed conditions, particularly in high-risk populations [23]. Promoting regular health check-ups and improving patient engagement with healthcare services are therefore critical strategies for reducing the burden of undiagnosed DM. Public health campaigns that encourage regular health screenings and raise awareness about the importance of early detection of chronic conditions could play a crucial role in reducing the burden of undiagnosed DM.

Despite the valuable insights provided by this study, it is essential to acknowledge its limitations. Firstly, the cross-sectional design restricts the ability to establish causal relationships between the identified factors and undiagnosed DM. Longitudinal studies are necessary to further explore these relationships, even though the identified associations are significant and do not imply causation. Secondly, the reliance on secondary data from the low-income registry introduces potential biases related to data accuracy and completeness. Despite efforts to ensure data quality during analysis, missing data or inaccuracies in the registry could affect the findings. Additionally, the study's focus on a specific population in a geographically isolated area may limit the generalisability of the findings to other populations. For future research, we recommend doing a comparison of similar studies in different places, like cities and rural mainland areas, to see how common undiagnosed DM is and what causes it in different groups of people. This study also underscores the need for more comprehensive and accessible screening programs, particularly in geographically isolated and low-income populations. Targeted health education campaigns should accompany these programs, raising awareness about the importance of regular health screenings and early diabetes detection. Integrating community-based approaches involving local stakeholders and healthcare providers could enhance the effectiveness of these interventions, ensuring they are culturally appropriate and sustainable in the long term.

## Conclusions

In conclusion, this study aimed to evaluate the prevalence, and factors associated with undiagnosed DM among the low-income population on Langkawi Island, Kedah, Malaysia. The findings revealed a significant prevalence of undiagnosed DM within this population, particularly among individuals with a higher BMI, those residing on smaller islands, and those lacking a documented medical history. These results underscore the critical need for enhanced screening and early detection programs tailored to address the unique challenges faced by geographically isolated and socio-economically disadvantaged communities. By identifying key predictors of undiagnosed DM, this study highlights the importance of targeted public health interventions that focus on increasing healthcare accessibility and promoting regular health screenings. The data produced in this study provides valuable insights that can guide the development of effective strategies to improve DM management and reduce the burden of undiagnosed DM in low-income and island populations.
